# Selection and Pilot Implementation of a Mobile Image Viewer: A Case Study

**DOI:** 10.2196/mhealth.4271

**Published:** 2015-05-27

**Authors:** Christine Marie Zwart, Miao He, Teresa Wu, Bart M Demaerschalk, Joseph Ross Mitchell, Amy K Hara

**Affiliations:** ^1^Medical Imaging InformaticsDepartment of RadiologyMayo ClinicScottsdale, AZUnited States; ^2^Department of Industrial EngineeringSchool of Computing, Informatics, and Decision Systems EngineeringArizona State UniversityTempe, AZUnited States; ^3^Neurocritical CareDepartment of NeurologyMayo ClinicPhoenix, AZUnited States

**Keywords:** mHealth, Pilot projects, Radiology, Telemedicine, Teleradiology

## Abstract

**Background:**

For health care providers, mobile image viewing increases image accessibility, which could lead to faster interpretation/consultations and improved patient outcomes.

**Objective:**

We explored the technical requirements and challenges associated with implementing a commercial mobile image viewer and conducted a small study testing the hypothesis that the mobile image viewer would provide faster image access.

**Methods:**

A total of 19 clinicians (9 radiologists, 3 surgeons, 4 neurologists, and 3 physician assistants) evaluated (1) a desktop commercial picture archiving and communication system (PACS) viewer, (2) a desktop viewer developed internally over 20 years and deployed throughout the enterprise (ENTERPRISE viewer) and (3) a commercial Food and Drug Administration class II-cleared mobile viewer compatible with Web browsers, tablets, and mobile phones. Data were collected during two separate 7-day periods, before and after mobile image viewer deployment. Data included image viewer chosen, time to view first image, technical issues, diagnostic confidence, and ease of use.

**Results:**

For 565 image-viewing events, ease of use was identical for PACS and mobile viewers (mean 3.6 for all scores of a possible 4.0), and significantly worse for the enterprise viewer (mean 2.9, *P*=.001). Technical issues were highest with the enterprise viewer (26%, 56/215) compared with the mobile (7%,19/259, *P*=.001) and PACS (8%, 7/91, *P*=.003) viewers. Mean time to first image for the mobile viewer (2.4 minutes) was significantly faster than PACS (12.5 minutes, *P*=.001) and the enterprise viewer (4.5 minutes, *P*=.001). Diagnostic confidence was similar for PACS and mobile viewers and worst for enterprise viewer. Mobile image viewing increased by sixfold, from 14% (37/269, before the deployment) to 88.9% (263/296, after the deployment).

**Conclusions:**

A mobile viewer provided faster time to first image, improved technical performance, ease of use, and diagnostic confidence, compared with desktop image viewers.

## Introduction

From educating to ordering tests, reporting, consulting, rounding, and sharing with patients, innovations in mobile technology are enhancing the way medicine is practiced in the 21st century. Emphasis has been placed on the availability of apps with the potential to benefit radiology residents [1,2] and staff [3,4]. The suggested toolbox of apps can contain eBooks, medical journals, note-taking apps, cloud data services, and audience polling tools, for example. The selection of a mobile image viewing solution, however, is much more complicated, requiring integration with picture archiving and communication systems (PACSs) and radiology information management systems, establishing secure logins, etc. As a result, incorporating mobile image viewers into the clinical context has been somewhat slow. Implementation has generally occurred for specific urgent care settings such as stroke or emergency medicine, rather than for general radiology use [5,6]. In this paper, we explored the technical requirements and challenges associated with implementing a commercial mobile image viewer and conducted a small study to test the hypothesis that the mobile image viewer would provide faster image access.

## Methods

### Selection

**Figure 1:**
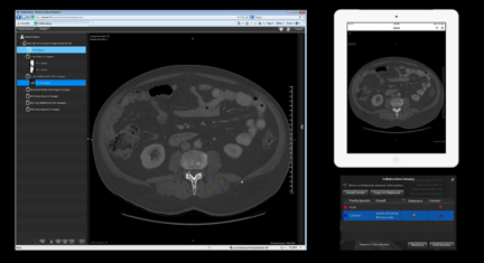
Overview of the ResMD interface and collaboration feature. A single ResMD session running simultaneously: in a desktop web browser (left) and as an application on an Apple iPad (top right). Interactions performed in either view are displayed interactively on both. Collaboration sessions are controlled via a collaboration window (bottom right). The collaboration window allows the “controller” to send invitations (via email), monitor who has joined the session, control the degree of interaction allowed by each user, and limit the display of patient information.

Several apps for diagnostic reading are currently available. Székely et al [3] identified 11 including ResolutionMD (ResMD, Calgary Scientific, Calgary, Canada) and Centricity Radiology Mobile Access and Siemens syngo.via WebViewer, now “ResolutionMD Mobile” (white-labeled versions of ResMD) [3]. The selection of ResMD for our pilot project was based on several factors, including the following: (1) Food and Drug Administration (FDA) class II clearance for diagnostic reads of all digital imaging and communication in medicine (DICOM) 3.0 imaging modalities (except mammography) on desktop Web browsers, iOS, and specific Android devices. FDA class II clearance indicates that the platform may be marketed for diagnostic use when traditional PACS workstations are not readily available. Each specific software, mobile device, and modality combination requires explicit class II clearance. (2) Vendor agnostic PACS connections—multiple PACS can be configured to connect simultaneously. (3) A client-server architecture with a “zero-footprint” client-side implementation. (4) Health Information Portability and Accountability Act compliance. In brief, this indicates that unencrypted protected health information is not stored on the mobile device after a viewing session terminates. (5) The ability to open specific series and examinations using a systematically built URL. The ability to systematically build URLs that simultaneously open the viewer and navigate to a specific series or examination has several utilities in research, conferences, and education. (6) Built-in features for advanced virtual collaboration. Advanced virtual collaboration utilities enable users to send an invitation link through email and have their viewing session streamed to both their device and that of the collaborators. All collaborators can scroll through images, window, and level, and point out features with visible cursors (Figure 1). (7) License costs were covered with a research agreement with Calgary Scientific, Inc (CSI). Additional information on the system architecture is provided in a previous study [6].

### Rationale

Those desiring a mobile image viewer included radiologists who are frequently on call, especially neuroradiologists and interventional radiologists, and wanted a faster and more reliable method to review cases for imaging consulting if not at the hospital or at home. In addition to radiologists, the stroke team, which included neurologists and neurosurgeons, desired to have rapid access to view acute head computed tomography scan images. The primary perceived benefit of a dedicated mobile image viewer was more rapid image access, which allows for faster communication of imaging findings, more rapid formation of treatment plans, leading to better outcomes and lower patient care costs.

The goal of our pilot implementation of a mobile image viewer was to collect data to determine whether long-term employment of such a mobile technology was warranted. We focused on quantifying the potential speed advantages of a mobile image viewing option and collecting clinician feedback on viewer performance and preferences.

### Institutional Review

We obtained Institutional Review Board approval to evaluate the chosen mobile image viewer (MOBILE) in comparison to our GE PACS workstations and a desktop viewer developed internally over 20 years and deployed throughout the enterprise (ENTERPRISE) [7].

Institutional policy mandating radiologists’ use of PACS during work hours (7 am to 6 pm weekdays, excluding holidays) was not modified for this study; radiologists recorded image access data after work, when any viewer could be used. All cases were officially interpreted and dictated by radiologists using the PACS workstation and digital dictation system. If preliminary reports were given by a radiologist using a mobile device, these were subsequently reviewed by the same radiologist on PACS for final interpretation. Any discrepancies noted by the dictating radiologist between the initial non-PACS and final interpretations were recorded.

### Evaluation

Our initial evaluation team of 19 clinicians included 9 radiologists (5 interventional radiologists and 4 neuroradiologists), 4 neurologists (all vascular neurologists and neurohospitalists), 3 physician assistants (2 orthopedic and 1 radiology), and 3 surgeons (2 neurosurgeons and 1 orthopedic surgeon). All participants had over 2 years’ experience with institutional desktop image viewers. The 4 neurologists collectively had viewed fewer than 50 cases using the mobile image viewer (as part of an independent telestroke pilot study). None of the other 15 clinicians had prior experience with the mobile image viewer. Before the implementation of the mobile image viewer, mobile access to images was accomplished using screen-sharing or remote-desktop apps.

Users manually recorded their radiology image viewer activity during two separate periods for 7 days each (before and after mobile viewer implementation) on a standardized datasheet. Preimplementation baseline data collection occurred when users could choose only between the two desktop viewers. Postimplementation data collection occurred 3 months after the implementation of the mobile viewer, when users could choose among the PACS desktop, ENTERPRISE, or a mobile viewer. Data collection focused on which of the 3 viewers was selected most often. Because institutional policy mandated the use of PACS during work hours (7 am to 6 pm weekdays, excluding holidays), radiologists recorded image access data after work, when any viewer could be used. Nonradiologist clinicians typically do not have access to PACS workstations, and thus, recorded all image access events both during and after work.

Data recorded for each image access event included date, time, location (inside or outside the hospital), device used (mobile or desktop), system used (PACS, ENTERPRISE, or mobile), time to first image, purpose of image access, and technical issues. For the self-selected viewer system, diagnostic confidence and ease of use were graded on a Likert-type scale (1, poor; 2, fair; 3, good; and 4, excellent). For each image event, data were recorded only for the chosen viewer. The same examination was not evaluated using other viewers.

The time to first image was recorded because it does not vary by examination type or complexity. It was defined as the time from when the clinician received a request to review images (ie, verbally or via a text page) to when the first image appeared on the screen. For desktop viewers, it included the time required to get to a workstation (including drive time, if necessary), to login to the workstation, and to display the first image. For the mobile viewer, it included time to login to the virtual private network, to launch the app, and to display the first image. Participants self-recorded time to first image using the stopwatch function on their telephone or wristwatch. An electronic survey was also distributed to participants at the end of the study period to collect data regarding their user experience.

XLSTAT (Addinsoft Inc, Brooklyn, NY, USA), a statistical analysis application for Microsoft Excel (Microsoft Inc, Redmond, WA, USA), was used to conduct the statistical testing. For all statistical tests, *P*<.05 was considered statistically significant. For continuous sample data (eg, access time), one-way analysis of variance was conducted to measure whether mean values differed significantly among viewers. When significant differences were found (ie, *P*<.05), the Tukey (honestly significant difference) method was used to conduct pairwise comparisons among the three systems. For ordinal sample data (eg, diagnostic confidence and ease of use, rated as 1, poor; 2, fair; 3, good; and 4, excellent), a Kruskal-Wallis test was conducted to determine whether measurements from the three viewers came from a single distribution. When significant differences (*P*<.05) were found, the Dunn method was used to conduct pairwise comparisons among the three systems. For nominal sample data (eg, technical issues, rated as 0, none; 1, difficulty logging in; 2, slow scrolling speed; 3, could not load all images; and 4, other), the chi-square test was conducted.

### Technical Details

Supporting the mobile image viewing frontend was a dedicated computer server running Red Hat Enterprise Linux 6.5 (RHEL6.5, Raleigh, NC, USA). It had two Intel Xeon E5-2643 processors (3.3 GHz, 4 physical, 8 virtual cores), 64-GB random access memory, 2-TB raided disk space, and two NVIDIA Quadro 6000 graphics cards.

Installation of the server requires a static Internet protocol address and an assigned hostname in the domain name system lookup table. Our server uses RHEL6.5 installed using a boot disk provided by CSI. Deploying the server is facilitated by RPM package manager and YellowdogUpdater, Modified.

For use within our hospital environment, the server was configured to connect to two systems, namely, a lightweight directory access protocol (LDAP) server to provide login authorization and a PACS. The LDAP and PACS systems were configured to accept this connection. These connections and configuration steps required participation and assistance from hospital information technology and radiology informatics staff.

We used a dedicated LDAP pool fed by the enterprise system to determine which hospital personnel had access to the mobile image viewing server without having to maintain usernames and passwords.

The DICOM standards facilitate configuring the server to communicate with PACS. The server functions as a DICOM network node and any PACS can be configured to allow query and move operations to it. Depending on institution procedures and preferences, the server can be configured to search the PACS directly for images based on patient name, patient ID, modality, scan date, and/or accession number. A c-move operation is used to pull images from PACS directly to the server random access memory. The server then performs rendering operations in response to user interactions on one or more client systems. The resulting two-dimensional images are then streamed interactively to one or more client devices (eg, tablets). In cases where the radiological reports are included in the PACS as a structured report object, the report will come through and be displayed as well. Alternatively, the server can be configured to pull reports from a Mitra reports broker (Mitra, Waterloo, ON, Canada) or using a plug-in to the Softek Illuminate reports interface (Softek, Prairie Village, KS, USA). It is also possible to run the viewer (ie, view images only) without a reports connection; we did this out of necessity for the first 2years of our pilot before implementing the Softek solution. Advanced users can use the Web or mobile interface to perform three-dimensional reconstructions, measurements, image markup, and screen captures, which can be pushed back to the PACS if your institution allows it. Our facility has chosen for the flow of images to be one way (from PACS).

## Results

### Clinical Experience

**Figure 2:**
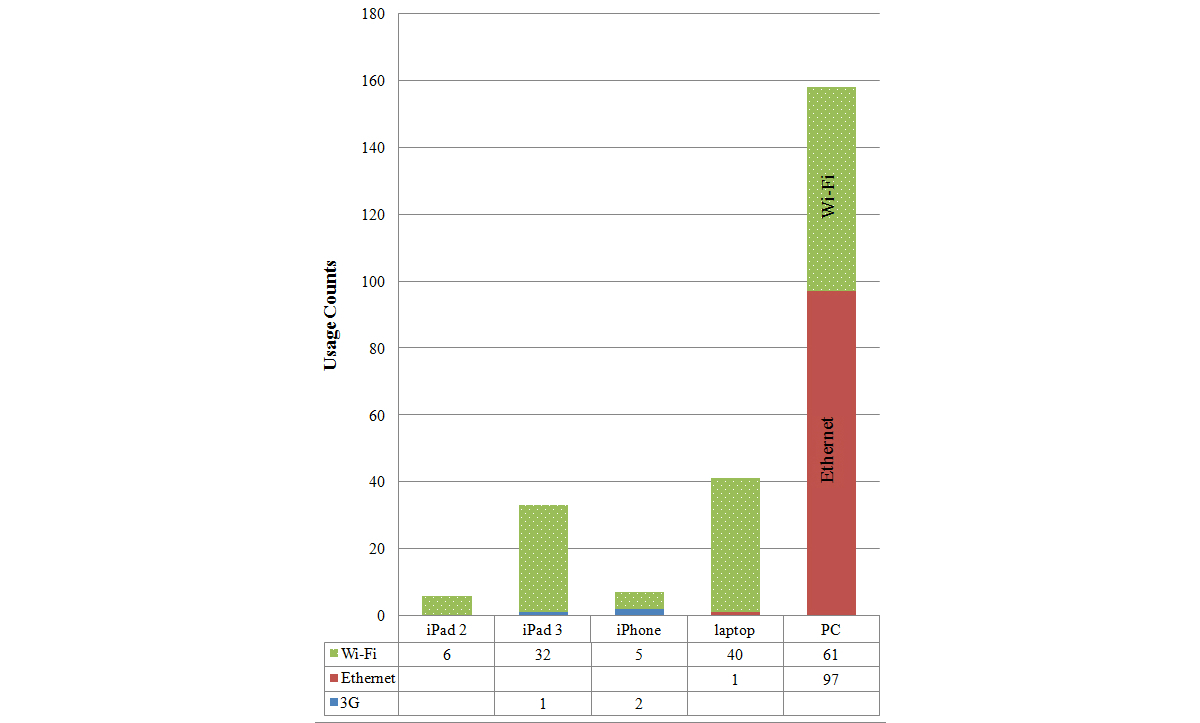
Usage counts are shown broken down by device and Internet connection type.

Data before and after mobile viewer implementation were collected from all 19 clinicians, for a total of 565 data points (269 preimplementation and 296 postimplementation): 259 using MOBILE, 215 using ENTERPRISE, and 91 using PACS viewers. Because radiologists collected data only when on call, most of their data points were collected outside the hospital (87.7%, 142/162). Most data from other clinicians were collected within the hospital (76.7%, 309/403) and during work hours (78.6%, 308/392). Mobile devices used were iPad2, iPad3, and iPhones, and Wi-Fi or 3G was used to connect to the Internet; desktop devices included laptops and clinical desktops (personal computers) connected via Ethernet or Wi-Fi. The relative device usage is shown in Figure 2.

Table 1 summarizes the scores for diagnostic confidence, ease of use, and overall technical issues by user group. Diagnostic confidence was rated good to excellent for all three viewing techniques but slightly higher scores were provided for PACS (mean 3.8), compared with mobile (mean 3.7) or ENTERPRISE (mean 3.4) viewers. The difference in diagnostic confidence between PACS and mobile viewers was not statistically significant (*P=*.08). Diagnostic confidence with ENTERPRISE was significantly lower (*P*=.001) than with the other two systems. No discrepancies were reported by radiologists between preliminary interpretations using the mobile viewer (n=71) and final interpretations on PACS. Preliminary interpretations were not rendered for other cases.

**Table 1:** Mobile radiology image viewers compared with conventional desktop viewers: qualitative results by user group.

		Data points, n	Diagnostic confidence	Ease of use	Fraction of cases with technical issues, n (%)
**All users**					
	PACS	91	3.8	3.6	7/91 (7.7)
	ENTERPRISE	215	3.4	2.9	56/215 (26.0)
	MOBILE	259	3.7	3.6	19/259 (7.3)
					
**Radiologists (n=9)**					
	PACS	89	3.8	3.6	7/89 (7.9)
	ENTERPRISE	2	3.0	3.0	0/2 (0.0)
	MOBILE	71	3.4	3.5	8/71 (11.3)
					
**Neurologists (n=4)**					
	PACS	0	N/A	N/A	N/A
	ENTERPRISE	29	3.6	3.4	6/29 (20.7)
	MOBILE	41	3.9	3.8	3/41 (7.3)
					
**Surgeons (n=3)**					
	PACS	0	N/A	N/A	N/A
	ENTERPRISE	43	3.0	2.6	27/43 (62.8)
	MOBILE	27	3.6	3.8	0/27 (0.0)
					
**Physicians Assistants (n=3)**					
	PACS	2	3.0	2.0	0/2 (0.0)
	ENTERPRISE	91	3.2	2.5	14/91 (15.4)
	MOBILE	120	3.6	3.5	8/120 (6.7)

The PACS and mobile viewers had identical ease-of-use ratings (mean 3.6), which were significantly superior to the ENTERPRISE rating (mean 2.9, *P*=.001). Technical issues were reported more frequently with CUSTOM (26%, 56/214) than with PACS (8%, 7/91, *P*=.003) or mobile viewers (7%, 19/259, *P*=.001). The mobile viewer also had significantly less frequent technical issues than PACS (*P*=.007). The most common technical complaints were slow scrolling speed through images (ENTERPRISE, 28/56), inability to load images (mobile, 11/19), and log-in problems (PACS, 5/7). All of these technical issues impeded the ability of the user to evaluate the study efficiently. Although slow scrolling was inefficient, it did allow examination review unlike the other issues.

The average time to first image was fastest with mobile viewers at 2.4 minutes (ENTERPRISE, 4.5 minutes and PACS, 12.5 minutes; Table 2). The average time to first image was significantly faster with mobile viewers, compared with PACS (*P*=.001) and ENTERPRISE (*P*=.001). ENTERPRISE, however, was significantly faster than PACS (*P*=.001). For the mobile viewer, the average time to first image remained less than 3 minutes, regardless of time of day or location. Time to first image for both ENTERPRISE and PACS was influenced by delays in getting to a usable workstation.

**Table 2:** Time to first image in minutes by type of image viewer.

Time/location	PACS	ENTERPRISE	MOBILE
All data points	12.5	4.5	2.4
Inside hospital	11.4	3.9^a^	2.7^b^
Outside hospital	12.7	7.9^a^	2.0^b^
During work hours (weekdays 7 am to 6 pm)	9.6	4.3^a^	2.2^b^
After work hours	12.9	5.3^a^	2.5^b^

^a^ *P*=.01 vs PACS

^b^ *P*<.01 vs PACS and ENTERPRISE custom viewer

The predominant purpose of image access before and after the implementation of mobile viewer was for decision making (67.6%, 200/296). Once the mobile viewer became available, image use for patient education increased from 18% (48/268) to 29% (86/296). Following its implementation, most patient education episodes were conducted with the mobile viewer (99%, 85/86).

At baseline, the most commonly used image viewer by nonradiologists was ENTERPRISE (180/182), whereas by radiologists, it was PACS (85/87). However, following its implementation, the mobile viewer became the most commonly used viewer by both nonradiologists (85.1%, 188/221) and radiologists (95%, 71/75). The use of mobile devices for image viewing increased more than sixfold from baseline to postimplementation (from 14%, 37/269, to 88.9%, 263/296, respectively).

Of the 19 users, 18 completed the poststudy survey (8 radiologists and 10 nonradiologists). Most users reported that they used the mobile viewer a few days each week (10/18, 56%). The remainder of users reported daily use (3/18, 17%), use only when on call (3/18, 17%), or rare or infrequent use (2/18, 11%). Overall, the mobile viewer was the preferred program for image viewing outside the hospital (11/18, 61%), preferred by more nonradiologists (7/10) than radiologists (4/8). Inside the hospital, nonradiologists preferred ENTERPRISE (5/10), whereas radiologists preferred PACS (7/8). Overall, the desire to permanently implement the mobile viewer was rated as moderate (n=7) or high (n=8) by most users (15/18, 83%), with the remaining users rating it as mild (n=2) or neutral (n=1). None of the respondents reported a preference to not implement the mobile image viewer (n=0).

## Discussion

### Clinical Experience

One of the major benefits we found was the two to six times faster time to first image (ie, 2-10 minutes faster) using the mobile image viewer, compared with either of the desktop programs. Time to first image was defined as the time from a request to review images to when the image first appeared on the screen. We focused on time to first image as a metric that would be useful to compare different systems and be consistent regardless of the examination size or complexity. The longer times with desktop viewers were likely due to the following two main factors: (1) desktop viewer access often required travel time to the hospital or home especially after work hours and (2) both desktop viewers, unlike the mobile viewer, simultaneously launched other programs (eg, dictation system), which consume time. The perceived benefits of more rapid image access included faster communication of imaging findings, more rapid formation of treatment plans, and improved outcomes leading to lower patient care costs.

Although the ability to quickly access images is important, image viewers must also provide high-quality images. Overall, the study demonstrated comparable diagnostic confidence between mobile and PACS viewers. In addition, although this study was not designed to address diagnostic accuracy, radiologists found no discrepancies in 71 studies interpreted with both mobile and PACS viewers. Further studies designed to directly assess the diagnostic quality of mobile viewing options in specific clinical contexts (eg, [8]) would be necessary before modifying diagnostic read protocols to include mobile options.

Since our initial trial period we have provided access to 277 users, comprising hospital staff and physicians including all radiologists and fellows. We make use of custom software to parse the system-generated log files to evaluate usage statistics by user, time of day, and day of week. We also continue to survey our user base both formally and informally.

### Technical Experience

Our experience with installing and integrating the back-end infrastructure necessary for a mobile image-viewing platform has been largely positive. We used hardware that closely resembles the high-end CSI-recommended servers and supports our current user base of nearly 500 (we generally have fewer than 5 simultaneous users). Servers can vary in price significantly (from US $5,000 to over US $15,000) depending on the number of simultaneous users accessing the system and whether or not advanced (three-dimensional) visualization capabilities will be enabled. The specific hardware needs of an organization would be based on the volume and intensity of the expected user base and may necessitate multiple servers.

The specific software installation and configuration process was relatively straightforward for a system administrator with basic Linux experience and greatly simplified by utilizing vendor suggestions for server configuration and operating system. Connectivity with enterprise and radiology informatics and computing services (LDAP and PACS) is essential.

Our PACS and RIS store images and reports separately, and as a result, our initial implementation did not include the radiology reports. Users consistently identified this as the largest shortcoming of the pilot. Our recent introduction of reports via the Softek interface has been an important step in increasing nonradiologist, nonemergent use of the app. By contrast, virtual collaboration is routinely touted as the largest benefit of the product (beyond rapid and mobile access) and is fully facilitated and enabled by the software. Maximizing the potential of this feature still requires active effort to integrate its use into the clinical routine.

### Challenges

Although the mobile image viewer is now available to all clinicians, it is still used by only a minority of the staff. We attribute this to several factors. One is the current lack of integration with more commonly used mobile apps such as the mobile electronic medical record (EMR). Having the mobile image viewer embedded into the mobile EMR would make it more easily accessible to users and not require them to have multiple apps open when evaluating the patient. In addition, the interface is different than the custom desktop interface used currently, requiring the user to learn a new method. Finally, even infrequent experiences of technical difficulties as significant as failing to load images (the most common issue seen with the mobile image viewer) are sufficient to sour users on use of the mobile image viewer in their clinical practice.

Based on our clinical demonstrations, younger users including residents and fellows seem more interested and less intimidated by this technology and we believe that focusing on trainees for more widespread use could be beneficial. In this way, the knowledge could travel “up” to more senior clinicians. Finally, user support is currently limited to a few people in our department. We are currently involved with efforts to have this technology accepted and supported by institutional resources, which could provide round-the-clock support.

### Future

We believe that mobile viewing technology with virtual collaboration technology has the potential to improve the speed and quality of care we deliver. Our future efforts are focused on integrating this system with the EMR and obtaining institutional support for more widespread implementation.

### Conclusions

The technical implementation and upkeep of the system are manageable but a significant and successful pilot or a roll out of this type of platform, or both, requires a dedicated team to train the user base and support workflow integration. Our initial clinical experiences suggest that user perceptions and quantifiable speed benefits afforded by a mobile image viewing option support the long-term adoption of such a platform.

## Abbreviations

CSI: Calgary Scientific, Inc
DICOM: digital imaging and communication in medicine
EMR: electronic medical record
FDA: Food and Drug Administration
LDAP: lightweight directory access protocol
PACS: picture archiving and communication system
ResMD: ResolutionMD
